# Characterization of the Lipidomic Profile of Human Coronavirus-Infected Cells: Implications for Lipid Metabolism Remodeling upon Coronavirus Replication

**DOI:** 10.3390/v11010073

**Published:** 2019-01-16

**Authors:** Bingpeng Yan, Hin Chu, Dong Yang, Kong-Hung Sze, Pok-Man Lai, Shuofeng Yuan, Huiping Shuai, Yixin Wang, Richard Yi-Tsun Kao, Jasper Fuk-Woo Chan, Kwok-Yung Yuen

**Affiliations:** 1 State Key Laboratory of Emerging Infectious Diseases, The University of Hong Kong, Pokfulam, Hong Kong Special Administrative Region, China; ybp1205@hku.hk (B.Y.); hinchu@hku.hk (H.C.); khsze@hku.hk (K.-H.S.); yuansf@hku.hk (S.Y.); rytkao@hku.hk (R.Y.-T.K.); 2 Department of Microbiology, Li Ka Shing Faculty of Medicine, The University of Hong Kong, Pokfulam, Hong Kong Special Administrative Region, China; u3005140@connect.hku.hk (D.Y.); vangor@hku.hk (P.-M.L.); shuaihp@connect.hku.hk (H.S.); jasyx@connect.hku.hk (Y.W.); 3 Carol Yu Centre for Infection, Li Ka Shing Faculty of Medicine, The University of Hong Kong, Pokfulam, Hong Kong Special Administrative Region, China; 4 Hainan-Medical University-The University of Hong Kong Joint Laboratory of Tropical Infectious Diseases, Hainan Medical University, Haikou 96708, China; 5 Hainan-Medical University-The University of Hong Kong Joint Laboratory of Tropical Infectious Diseases, The University of Hong Kong, Pokfulam, Hong Kong Special Administrative Region, China; 6 The Collaborative Innovation Center for Diagnosis and Treatment of Infectious Diseases, The University of Hong Kong, Pokfulam, Hong Kong Special Administrative Region, China

**Keywords:** lipidomics, UHPLC–MS, HCoV-229E, MERS-CoV

## Abstract

Lipids play numerous indispensable cellular functions and are involved in multiple steps in the replication cycle of viruses. Infections by human-pathogenic coronaviruses result in diverse clinical outcomes, ranging from self-limiting flu-like symptoms to severe pneumonia with extrapulmonary manifestations. Understanding how cellular lipids may modulate the pathogenicity of human-pathogenic coronaviruses remains poor. To this end, we utilized the human coronavirus 229E (HCoV-229E) as a model coronavirus to comprehensively characterize the host cell lipid response upon coronavirus infection with an ultra-high performance liquid chromatography-mass spectrometry (UPLC–MS)-based lipidomics approach. Our results revealed that glycerophospholipids and fatty acids (FAs) were significantly elevated in the HCoV-229E-infected cells and the linoleic acid (LA) to arachidonic acid (AA) metabolism axis was markedly perturbed upon HCoV-229E infection. Interestingly, exogenous supplement of LA or AA in HCoV-229E-infected cells significantly suppressed HCoV-229E virus replication. Importantly, the inhibitory effect of LA and AA on virus replication was also conserved for the highly pathogenic Middle East respiratory syndrome coronavirus (MERS-CoV). Taken together, our study demonstrated that host lipid metabolic remodeling was significantly associated with human-pathogenic coronavirus propagation. Our data further suggested that lipid metabolism regulation would be a common and druggable target for coronavirus infections.

## 1. Introduction

Coronaviruses are enveloped viruses with a large single-strand, positive-sense RNA genome [1,2]. As of today, there are a total of six coronaviruses that are known to infect humans, including human coronavirus OC43 (HCoV-OC43), human coronavirus 229E (HCoV-229E), severe acute respiratory syndrome coronavirus (SARS-CoV), human coronavirus HKU1 (HCoV-HKU1), human coronavirus NL63 (HCoV-NL63), and the Middle East respiratory syndrome coronavirus (MERS-CoV) [3]. These human-pathogenic coronaviruses cause a broad range of clinical manifestations. HCoV-OC43, HCoV-229E, HCoV-HKU1, and HCoV-NL63 cause mild, self-limiting upper respiratory tract infections. In contrast, SARS-CoV and the recently emerged MERS-CoV may cause severe pneumonia with acute respiratory distress syndrome, multi-organ failure, and death in both immunocompetent and immunocompromised hosts [4,5,6,7].

Lipids play crucial roles at various stages in the virus life cycle. First, lipids can serve as the direct receptors or entry co-factors for enveloped and non-enveloped viruses at the cell surface or the endosomes [8,9]. Second, lipids and lipid synthesis play important roles in the formation and function of the viral replication complex [10,11]. Third, lipid metabolism can generate the required energy for efficient viral replication [12]. Moreover, lipids can dictate the proper cellular distribution of viral proteins, as well as the trafficking, assembly, and release of virus particles [13,14]. In this regard, the host lipid biogenesis pathways play indispensable roles in modulating virus propagation.

As in other viruses, lipids play key roles in the life cycle of coronaviruses. Coronaviruses confiscate intracellular membranes of the host cells to generate new compartments known as double membrane vesicles (DMVs) for the amplification of the viral genome. DMVs are membranous structures that not only harbor viral proteins but also contain a specific array of hijacked host factors, which collectively orchestrate a unique lipid micro-environment optimal for coronavirus replication [15]. A recent study indicated that a key lipid processing enzyme, cytosolic phospholipase A2α enzyme (cPLA2α) that belongs to the phospholipase A2 (PLA2) superfamily, was closely associated with DMVs’ formation and coronaviruses’ replication [16]. The viral protein and RNA accumulation, as well as the production of infectious virus progeny, were significantly diminished in the presence of cPLA2α inhibitor [16]. At the same time, phospholipase A2 group IID (PLA2G2D), an enzyme that predominantly contributes to anti-inflammatory/pro-resolving lipid mediator expression, contributed to worsened outcomes in mice infected with SARS-CoV by modulating the immune response [17]. However, to date, the change and modulating effects of the specific lipids involved in lipid rearrangement upon coronavirus infection remains largely unexplored.

To obtain a comprehensive and unbiased profile of perturbed lipids upon coronavirus infection, we performed mass spectrometry (MS)-based lipidomics profiling on coronavirus-infected cells using HCoV-229E as a model virus. Specific lipids including glycerophospholipids and fatty acids (FAs) upon virus infection were identified, which represented the lipid species that were rearranged by HCoV-229E infection. Further pathway analysis revealed that the linoleic acid (LA) and arachidonic acid (AA) metabolism axis was the most perturbed pathway upon HCoV-229E infection. Importantly, supplement of additional LA and AA to coronavirus-infected cells significantly inhibited virus replication of both HCoV-229E and the highly virulent MERS-CoV, suggesting that the LA–AA metabolism axis is a common and essential pathway that could modulate coronavirus replication. In this regard, temporal modulation of the host lipid profile is a potential novel strategy to combat emerging human coronaviruses.

## 2. Materials and Methods

### 2.1. Materials

High performance liquid chromatography (HPLC)-grade methanol, acetonitrile, chloroform and 2-propanol were purchased from Merck (Darmstadt, Germany). HPLC-grade water was prepared using a Milli-Q water purification system (Millipore, Burlington, MA, USA). Analytical grade acetic acid and commercial standards used for biomarker identification were purchased from Sigma-Aldrich (St. Louis, MO, USA). Internal standards (IS) including Arachidonic acid-d8, 15(S)-HETE-d8, Leukotriene-B4-d4 and Platelet-activating factor C-16-d4 (PAF C-16-d4) were purchased from Cayman Chemical (Ann Arbor, MI, USA) [18].

### 2.2. Viruses and Cells

Huh-7 and VeroE6 cells were maintained in Dulbecco’s modified Eagle medium (DMEM) supplemented with 10% heat-inactivated fetal bovine serum (FBS), 100 U/mL penicillin, and 100 g/mL streptomycin (5% CO_2_ at 37 °C). MERS-CoV (EMC/2012 strain) was kindly provided by Professor Ron Fouchier (Erasmus Medical Center, Rotterdam, The Netherlands). MERS-CoV and HCoV-229E were cultured in VeroE6 cells in serum-free DMEM supplemented with 100 U/mL penicillin and 100 g/mL streptomycin as we described previously [19,20,21]. The supernatants were harvested when cytopathic effects (CPE) were observed and centrifuged to generate the viral stocks. The viral stocks were titrated by plaque assay on VeroE6 cells and stored at −80 °C as previously described [22,23]. Briefly, confluent VeroE6 cells were infected with 10-fold serial viral dilutions. The cells were incubated with diluted viruses at 37 °C for 1 h and subsequently overlaid with 1% low-melting-point agarose (Promega, Madison, WI, USA). The cells were fixed with 4% formaldehyde as the plaques were observed and then stained with 0.2% crystal violet. All experiments involving live MERS-CoV followed the approved standard operating procedures of the biosafety level 3 facility as previously described [24,25,26,27].

### 2.3. Lipid Treatment of Middle East Respiratory Syndrome Coronavirus (MERS-CoV)-Infected and Human Coronavirus (HCoV-229E)-Infected Huh-7 Cells

Huh-7 cells were seeded into 24-well plate to reach 90% confluency and infected with MERS-CoV or HCoV-229E at multiplicity of infection (MOI) of 0.005 or 1, respectively. After 1 h of inoculation, the cells were washed with phosphate-buffered saline (PBS) and maintained in lipids-supplemented medium at the indicated concentrations for 24 h. AA, LA, oleic acid (OA), and palmitic acid (PA) were dissolved in ethanol and ethanol was used as a negative control. The lipids were purchased from Cayman Chemical (Ann Arbor, MI, USA). The supernatants and cell lysates were collected at 24 h post-infection. The viral genome copy numbers were determined by reverse-transcription quantitative polymerase chain reaction (RT-qPCR) as previously described [28,29,30].

### 2.4. Lipid Extraction for Lipidomics Profiling

Confluent Huh-7 cells were mock infected or infected with HCoV-229E at MOI of 1 and incubated in DMEM medium. At 24 hpi, cells were collected for cellular lipid extraction. The lipid extraction was performed for liquid chromatography-mass spectrometry (LC-MS) analysis according to a previously described protocol with slight modifications [31,32]. Inactivation of virus infectivity was confirmed before further processing as we previously described with some modifications [33]. Briefly, 500 µL of ice-cold 150 mM ammonium bicarbonate solution was added to dissociate cells. Two millilitres of chloroform/methanol (*v*/*v* 2:1) containing IS were added, followed by vortexing and centrifugation at 4500 rpm for 10 min at 4 °C. The bottom phase was transferred to glass vials and dried using a vacuum concentrator for storage at −80 °C. The dried samples were reconstituted in 250 µL solvent mixture containing methanol/2-propanol/water (*v*/*v*/*v* 5:4:1) for LC-MS analysis. After centrifugation at 14,000 rpm for 10 min at 4 °C, supernatants were transferred to LC vials for LC-MS analysis.

### 2.5. Ultra-High Performance Liquid Chromatography-Electrospray Ionization-Quadrupole-Time of Flight-Mass Spectrometry (UPLC-ESI-Q-TOF-MS) Analysis

The lipid extract was analyzed using an Acquity UPLC system coupled to a Synapt G2-Si High Definition Mass Spectrometry (HDMS) system (Waters Corp., Milford, MA, USA). The chromatography was performed on a Waters ACQUITY BEH C18 column (1.7 μm, 2.1 × 100 mm, I.D., 1.7 mm, Waters Corp., Milford, MA, USA). The mobile phase consisted of (A) 0.1% acetic acid in water and (B) acetonitrile. Gradient elution applied for ultra-high performance liquid chromatography-mass spectrometry (UPLC-MS) analysis was described in Table S1. The column and autosampler temperature were maintained at 45 °C and 4 °C, respectively. The injection volume was 5 µL [34]. 

The mass spectral data were acquired in both positive and negative modes. The capillary voltage, sampling cone voltage and source offset were maintained at 2.5 kV, 60 V, and 60 V, respectively. Nitrogen was used as desolvation gas at a flow rate of 800 L/h. The source and desolvation temperatures were maintained at 120 °C and 400 °C, respectively. Mass spectra were acquired over the *m*/*z* range of 50 to 1200. The SYNAPT G2-Si HDMS system was calibrated using sodium formate clusters and operated in sensitivity mode. Leucine enkephalin was used as a lock mass for all experiments. MS/MS acquisition was operated in the same parameters as MS acquisition. Collision energy was applied at the range from 20 to 40 eV for fragmentation to allow putative identification and structural elucidation of the significant lipids.

### 2.6. Data Processing and Statistical Data Analysis

Acquisition of the raw data was performed using MassLynx software version 4.1 (Waters Corp., Milford, MA, USA) and raw data were converted to the common data format (NetCDF) files using conversion software Databridge (Waters Corp., Milford, MA, USA). The NetCDF data were subsequently deconvolved into a usable data matrix using the XCMS software (http://metlin.scripps.edu/download/) [35] and the grouping of features was performed using the CAMERA R package [36]. Preprocessed data were then exported as a .csv file for further data statistical analysis. MetaboAnalyst 3.0 (http://www.metaboanalyst.ca) and SIMCA-P V12.0 (Umetrics, Umeå, Sweden) were used for univariate and multivariate statistical analysis, respectively [37]. For univariate analysis, statistical significance of features was determined between the mock and HCoV-229E infected group using the Student’s *t*-test and fold change. The *p*-value < 0.05 and fold change > 2 were used as criteria for significant features selection. For multivariate analysis, the features were subjected to Pareto scaling firstly then orthogonal partial least squares discriminant analysis (OPLS-DA) was performed as a supervised method to find important variables with discriminative power. The OPLS-DA model was evaluated with the relevant R2 and Q2. The variable importance in projection (VIP), which reflects both the loading weights for each component and the variability of the response explained by this component, was used for feature selection [38].

### 2.7. Lipids Identification

MS/MS fragmentation was performed on the significant features with high abundances. The significant features identification were carried out by searching accurate MS and MS/MS fragmentation pattern data in the METLIN database (Metabolomics Database, http://metlin.scripps.edu/), Human Metabolome Database (http://www.hmdb.ca/), and LIPD MAPS (Lipidomics Gateway, http://www.lipidmaps.org/). For confirmation of lipid identity using authentic chemical standard, MS/MS fragmentation pattern of the chemical standard was compared with that of candidate lipid under the same LC-MS condition to reveal any matching [18,39].

## 3. Results

### 3.1. Omics-Based Statistical Analysis for Significant Features

To investigate how coronavirus perturbs host lipid metabolism, we performed lipidomics analysis on HCoV-229E-infected Huh7 cells and compared the results with those of the mock-infected cells. The preliminary features list included precursor ions, adducts and isotope ions, which were imported into the MetaboAnalyst and SIMCA-P software for further analysis. The R2X/ R2Y, represented the X/Y variables explanation rate of the OPLS-DA model, were 83.0% and 98.8%, respectively. The predicted component, as estimated by cross-validation, was 0.97 (Q2). These cross-validated parameters were satisfactory for OPLS-DA mode (Supplementary Figure S1a). At the same time, the permutation test (100 times) also indicated that the validated model was satisfied (Supplementary Figure S1b). Overall, our results demonstrated that these significant lipid features could be selected by the validated statistical model for subsequent identification.

### 3.2. Identification of Lipids Specific to HCoV-229E

**Figure 1:**
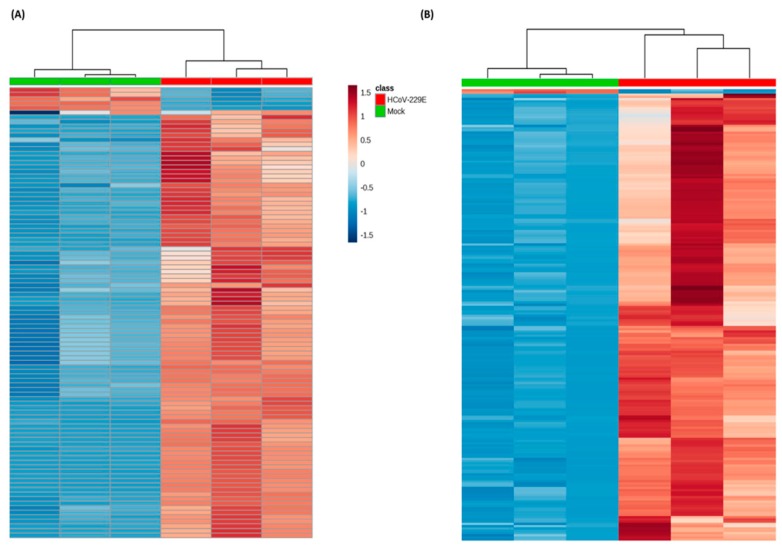
Heatmap showing the lipidomic analysis of human coronavirus 229E (HCoV-229E)-infected versus non-infected Huh-7 cells. Each rectangle represents an ion feature colored by its normalized intensity scale from blue (decreased level) to red (increased level). The dendrogram on the top was constructed based on the lipid intensity (similarity measure using Euclidean, and the Ward clustering algorithm). HCoV-229E, HCoV-229E-infected cells; Mock, non-infected cells. (**A**) Significant ion features in negative detection mode; (**B**) significant ion features in positive detection mode.

**Figure 2:**
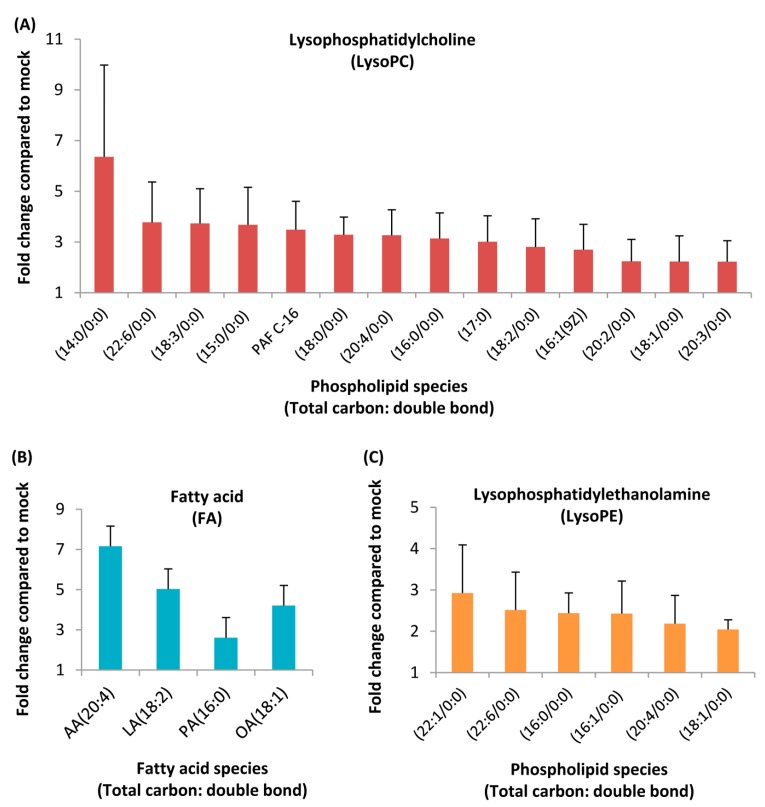
Liquid chromatography-mass spectrometry (LC/MS) analysis of HCoV-229E-infected cells revealed a homeostatic change in lipid levels. Huh-7 cells were mock- or HCoV-229E-infected and harvested at 24 hpi. The peak heights of these lipids were calculated and the fold change plotted with GraphPad Prism 5. (**A**) Lysophosphatidylcholine (LysoPC), (**B**) fatty acid (FA), (**C**) lysophosphatidylethanolamine (LysoPE). AA, arachidonic acid; LA, linoleic acid; PA, palmitic acid; OA, oleic acid.

**Figure 3:**
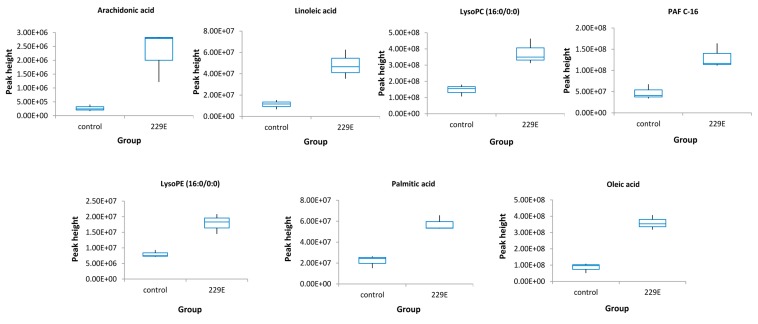
Box-whisker plots of the 7 standard confirmed lipids that were distinguished between the HCoV-229E-infected samples and the non-infected controls. The peak height was generated by LC-MS raw data. Control, non-infected cells; 229E, HCoV-229E-infected cells.

A total of 206 (positive mode) and 100 (negative mode) ion features were selected according to the omics-based statistical analysis method. These ion features were significantly discriminative between HCoV-229E-infected and mock-infected cells. To observe the discrimination trend in more detail, a hierarchical clustering analysis was performed based on the degree of similarity of lipid abundance profiles to show the overview trend of all significant ion features. As indicated in Figure 1, most of the significant features from both negative mode (Figure 1A) and positive mode (Figure 1B) expressed an up-regulation trend after HCoV-229E infection compared with the mock infection controls. Furthermore, to identify lipids specific to HCoV-229E infection, these significant features were grouped and annotated using the CAMERA software, and the potential precursor ions were used to perform further MS/MS experiments for obtaining their fragmentation patterns. Finally, a total of 24 lipids were identified, which could be classified into three lipid classes, including lysophosphatidylcholine (lysoPC), lysophosphatidylethanolamine (lysoPE) and fatty acid (FA). The chromatogram peak heights of these identified lipids were generated by LC-MS raw data and the ratio between infected and mock-infected cells was determined. As demonstrated in Figure 2, we found a consistent up-regulation trend of the identified lipids in HCoV-229E-infected cells. In particular, lysoPC was the predominant lipid class of all identified, accounting for approximately 60% of all identified lipids with significant elevation (Figure 2A). At the same time, arachidonic acid (AA), which belongs to the FA class, showed the highest increase in fold-change among all identified lipids with a maximum of 7.1-fold increase (Figure 2B). In addition, the level of lysoPEs (Figure 2C) was also up-regulated with a maximum fold change of 2.93, which was comparatively less than that of the lysoPCs and FAs. The identities of lysoPC (16:0/0:0), platelet-activating factor C-16 (PAF C-16), lysoPE (16:0/0:0), AA, LA, PA and OA were confirmed by matching the retention time (RT) and MS/MS fragmentation patterns of the authentic chemical standards that distinguish between HCoV-229E-infected cases and non-infected controls (Figure 3). The detailed information of the 24 identified lipids was listed in Table 1. MS/MS fragmentation patterns of five representative lipids and corresponding standards are also demonstrated in Supplementary Figure S2.

### 3.3. Pathway Analysis of HCoV-229E-Infected Huh7 Cells

**Figure 4:**
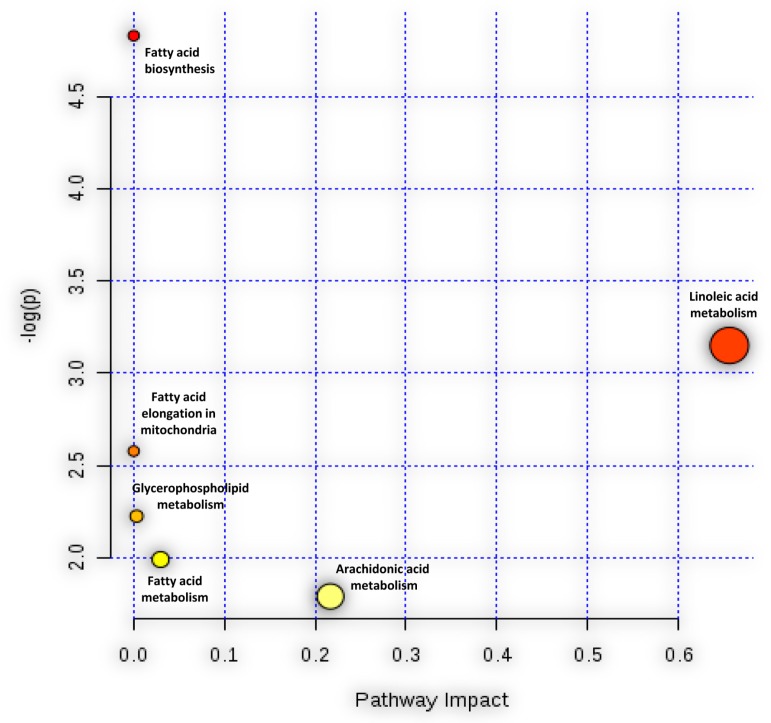
Pathway analysis associate with HCoV-229E infection was carried out by MetaboAnalyst. The *Y*-axis, “log(p)”, represented the transformation of the original *p*-value calculated from the enrichment analysis. The *X*-axis, “Pathway Impact”, represented the value calculated from the pathway topology analysis.

Based on the list of significantly up-regulated lipids after HCoV-229E-infection, MetaboAnalyst (http://www.metaboanalyst.ca) was applied to investigate which pathway might be markedly perturbed. The result of the pathway analysis was graphically presented in Figure 4. From the enrichment analysis results, the LA metabolism pathway and FA biosynthesis pathway had a statistically significant raw *p*-value (raw *p* < 0.05, as shown in the *Y*-axis). Pathway impact results indicated that the LA metabolism and AA metabolism pathways presented higher impact than the other pathways, as indicated in the *X*-axis value. Combining the above two analysis results, we postulated that the LA metabolism pathway to be a markedly perturbed pathway that correlated with the lipid rearrangement process induced by HCoV-229E infection.

**Figure 5:**
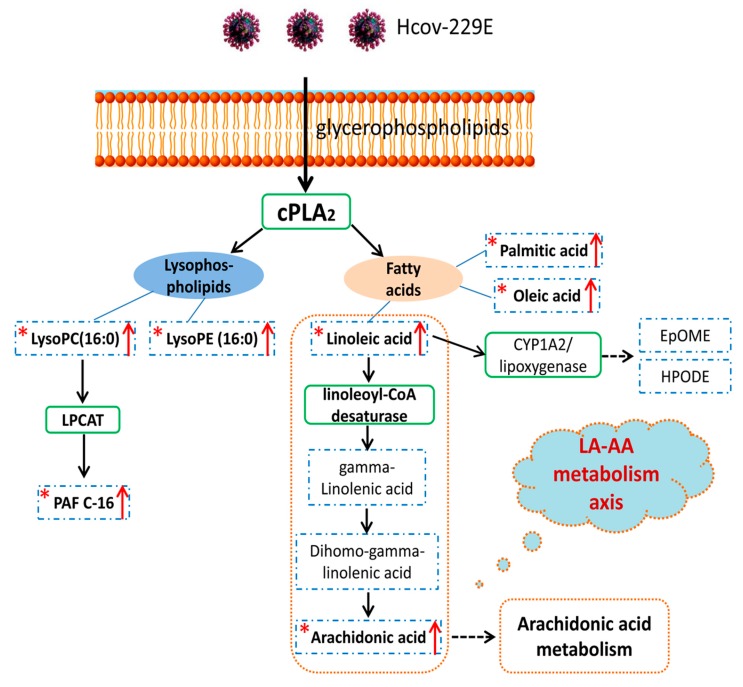
The pathway map based on identified lipids and linoleic acid metabolism recorded in the Kyoto Encyclopedia of Genes and Genomes (KEGG) PATHWAY Database. The star mark “*” indicates the lipids could be matched with commercial standards and have an up-regulation trend. The red arrow represents the up-regulation trend. The blue dashed rectangle and green solid rectangles represent lipids and corresponding enzyme in this pathway, respectively. The orange dashed line represents the LA–AA metabolism axis.

To better understand the current pathway analysis results and the cellular lipid signaling response upon HCoV-229E infection, we constructed a global LA pathway map based on the pathway information in the Kyoto Encyclopedia of Genes and Genomes (KEGG) database (https://www.genome.jp/kegg/) and literature mining (Figure 5). Upon HCoV-229E infection, the glycerophospholipids, as main components of the cell membrane, were metabolized to lysophospholipids and FAs after cPLA2 enzyme activation. Lysophospholipids such as lysoPCs and lysoPEs were correspondingly increased after HCoV-229E infection. Moreover, lysoPCs could be further metabolized to platelet-activating factor. FAs were also released from glycerophospholipids but only LA and AA could initiate downstream pathways to generate corresponding metabolites. The up-regulation of both lysophospholipids and FAs were partially confirmed by authentic standards. Furthermore, to investigate the downstream pathways trend of FAs, the authentic standards were also applied in LC-MS method to confirm whether these downstream lipids were changed correspondingly. As illustrated in Figure 5, AA is a downstream lipid of LA and the origin lipid of AA metabolism pathway. The identity of AA was confirmed by authentic standard (Supplementary Figure S2D), which was found to be significantly up-regulated. Therefore, combining pathway analysis and the authentic standards verification results, our data suggested that the LA–AA metabolism axis was the most significantly perturbed pathway and might be associated with lipids rearrangement or other processes in HCoV-229E infection.

### 3.4. Lipids Treatment of Virus-Infected Cells

**Figure 6:**
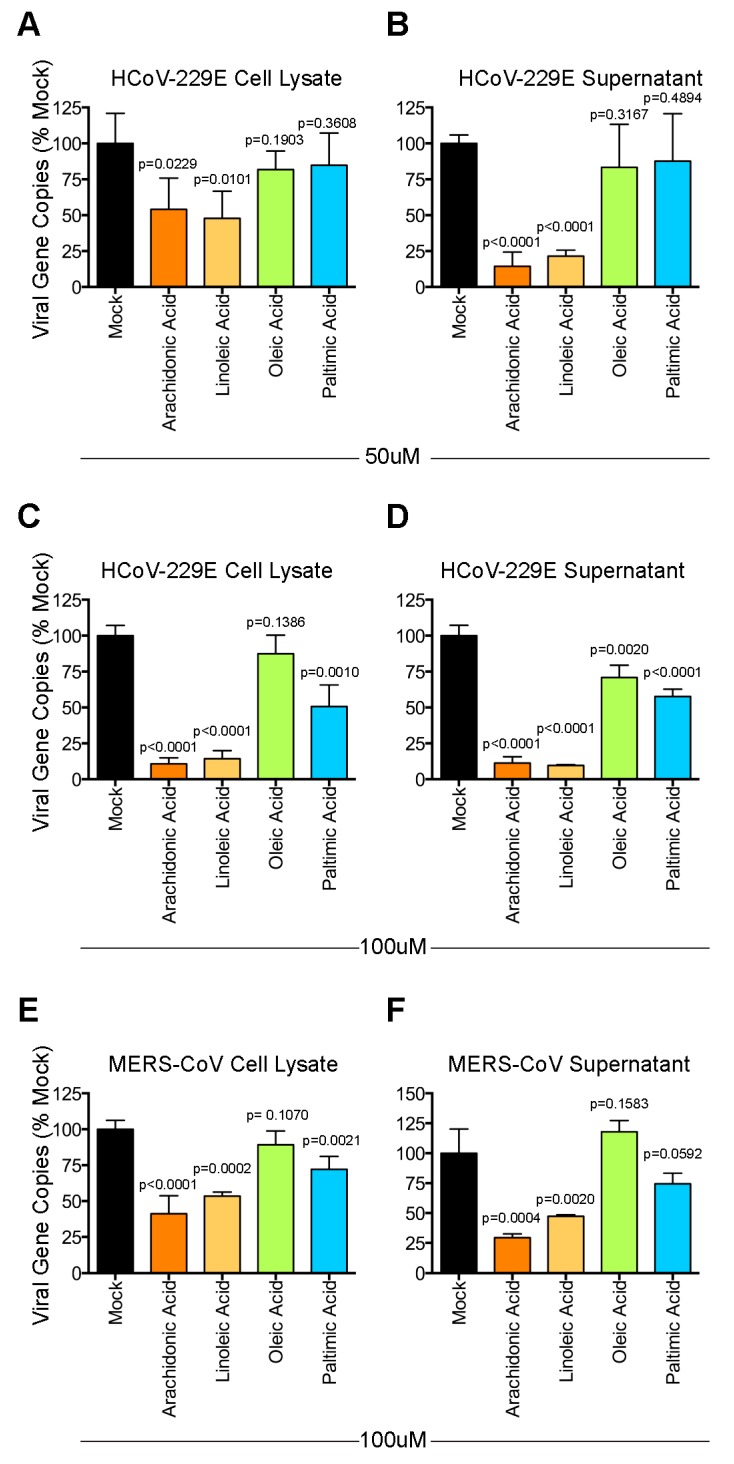
Modulatory effect of lipids on HCoV-229E and Middle East respiratory syndrome coronavirus (MERS-CoV). Huh-7 cells were infected with HCoV-229E. After 1 h of inoculation, the virus inoculum was replaced with medium containing 50 µM (**A**,**B**) or 100 μM (**C**,**D**) of lipids and incubated for 24 h. The supernatants and cell lysates were collected for reverse-transcription quantitative polymerase chain reaction (RT-qPCR) analysis. In parallel, Huh-7 cells were infected with MERS-CoV. After 1 h of inoculation, the virus inoculum was replaced with medium containing 100 μM (**E**,**F**) of lipids and incubated for 24 h. The supernatants and cell lysates were collected for RT-qPCR analysis. Statistical significance was determined by Student’s t-test by comparing the individual lipid-treated group with the mock-treated group (*n* = 4). The difference was considered significant when *p* < 0.05.

To investigate the potential implication of the perturbed LA-AA metabolism axis in HCoV-229E infection, we treated HCoV-229E-infected Huh7 cells with LA and AA and included PA and OA for comparison. The LA and AA were mapped and played a vital role in the perturbed LA-AA metabolism axis (Figure 5). In contrast, PA and OA were not mapped in the perturbed pathway and may only be produced from glycerophospholipids due to cPLA2 enzyme activation. Huh-7 cells were infected with HCoV-229E and treated with AA, LA, PA, or OA. The cell lysates and culture supernatants were harvested at 24 h post-infection to determine the viral genome copy number by RT-qPCR. As shown in Figure 6, LA and AA consistently inhibited the replication of HCoV-229E as evidenced by the decrease in virus genome copies in both cell lysate (Figure 6A,C) and supernatant samples (Figure 6B–D). In contrast, PA inhibited HCoV-229E replication only when supplied at high concentration while HCoV-229E replication was largely independent of OA (Figure 6A–D).

To further investigate if the modulatory effects of LA and AA were conserved among other human-pathogenic coronaviruses, we evaluated the effects of these lipids on the replication of the recently emerged and highly virulent MERS-CoV. Our data demonstrated that LA and AA potently suppressed MERS-CoV replication in a similar manner as HCoV-229E (Figure 6E,F). Overall, our results demonstrated that exogenously supplied LA and AA could interfere with the optimal replication of human-pathogenic coronaviruses, which suggested that the LA–AA metabolism axis was significantly involved in the propagation of these viruses.

## 4. Discussion

In this study, a MS-based lipidomics approach was established to characterize the host cell lipid changes upon coronavirus infection. Univariate and multivariate statistical analyses were applied in data processing for the selection of significant lipid features. A total of 24 lipids including lysophospholipids and FAs were identified and were consistently up-regulated in HCoV-229E-infected cells. Seven representative lipids were confirmed by authentic standards, including lysoPC (16:0/0:0), PAF C-16, lysoPE (16:0/0:0), AA, LA, PA and OA. Subsequent pathway analysis indicated that the LA–AA metabolism axis, consisting of LA and AA as important precursor lipids, was substantially perturbed after HCoV-229E infection. Moreover, we demonstrated that exogenously supplied LA and AA were capable of inhibiting the replication of HCoV-229E and the highly pathogenic MERS-CoV, which suggested the LA–AA metabolism axis to be a conserved and essential pathway in the propagation of human coronaviruses.

A total of 24 lipids including lysoPCs, lysoPEs and unsaturated/saturated FAs were identified to be significantly upregulated after HCoV-229E infection. Twenty of these 24 (83.3%) lipids were lysoPC and lysoPE. LysoPC is the most abundant lysophospholipid in humans, with a high plasma concentration of several hundred micromoles. In addition, lysoPC was a potent inhibitor and could reversely arrest pore expansion during syncytium formation mediated by diverse viral fusogens [40]. Another lysophospholipid, lysoPE, is present at low concentrations in vivo but they induce various cellular responses such as activation of mitogen-activated protein kinase (MAPK) and neuronal differentiation when applied to cells in vitro [41]. Among the identified FAs, the LA and AA both belong to polyunsaturated omega-6 fatty acid and are essential fatty acids. In addition, LA is the metabolic precursor of AA, both of which are key components of the cell membrane. LA and AA also play fundamental roles in the biological function of many tissues by modulating enzymes, ion channels, receptors, as well as inflammation [42].

Coronavirus replication is associated with intracellular membrane rearrangement and depends on the formation of double membrane vesicles (DMVs) and other membranous structures as replicative organelles [16]. The cell membrane components consist mainly of glycerophospholipid components such as phosphatidylcholine (PC), phosphatidylethanolamine (PE), lysophosphatidylcholine (lysoPC), and lysophosphatidylethanolamine (lysoPE). A specific phospholipids composition is required by different viruses to form the optimal replicative organelles best suited for their replication [43]. Moreover, the lysoPC/PE was produced from PC/PE by cPLA2 activation, which simultaneously generated corresponding fatty acid moiety. In this regard, cPLA2 activation is commonly believed to be beneficial for virus replication [16,17].

In our study, we found that a number of lysophospholipids and FAs downstream of cPLA2 activation, were upregulated upon HCoV-229E infection. The upregulation of these lipid species including LA and AA were believed to promote efficient coronavirus replication. However, when we evaluate this hypothesis by exogenously supplementing additional LA and AA to HCoV-229E- or MERS-CoV-infected cells, we noticed a significant reduction in virus replication. Taken together, our data suggested that coronavirus infection did not randomly perturb the cellular lipid compositions. Instead, we speculate that coronaviruses precisely modulate and rearrange the host lipid profile to reach an intricate homeostasis optimized for its replication. Any exogenous manipulation that disrupts the equilibrium may interfere with the optimal replication of the viruses. Alternatively, supplementing LA and AA might disturb the LA–AA metabolism axis and result in feedback reversion of lysophospholipids into phospholipids through Land’s cycle [44], thus limiting virus replication. 

In addition, LA and AA are polyunsaturated fatty acids that are biological signaling precursors. They can be metabolized to important eicosanoids and metabolites, which play multiple roles in the host immune response and the pathogenesis of viral infections [45,46,47]. However, previous study had suggested that arachidonic acid (AA) downstream metabolites show no evidence of anti-coronaviral activity as observed through special inhibitors of cyclooxygenases (COX) 1/2 and 5-lipoxygenase (LOX), which are two key enzymes requiring AA as a precursor. The results indicated AA downstream products may not have a significant effect on coronaviruses replication, at least in vitro [16,48]. In this regard, the function of the downstream metabolites of LA and AA may play key roles in the pathogenesis of coronaviruses in vivo.

## 5. Conclusions

In the present study, we revealed that the cellular lipid profile was rearranged upon HCoV-229E infection. A total of 24 lipids including lysoPCs, lysoPEs and FAs were upregulated. Among them, LA and AA, which were mapped into the LA-AA metabolism axis, demonstrated strong modulatory effects on the replication of HCoV-229E and the highly pathogenic MERS-CoV. In this regard, our data suggested that optimal coronavirus replication required a specific composition of cellular lipids and any disruption could decrease the efficiency of coronavirus replication. Thus, the MS-based lipidomics strategy could be used to monitor virus-specific lipid requirement, to discover the perturbed pathways and identify novel lipids to interfere with virus replication. In further studies, combining lipidomics data with biological and immunological data may help to elucidate specific pathogenic mechanisms and identify novel treatment strategies for virus infections.

## Figures and Tables

**Table 1:** The 24 lipids that were significantly different between HCoV-229E-infected and mock-infected samples.

Significant Lipids	Trend in HCoV-229E vs. Control	Molecular Formula	Detection Mode	Retention Time	Accurate Mass in Detection Mode	Fold Change	*p*-Value	VIP
lysoPC(16:0/0:0)^S^	up-regulation	C24H50NO7P	pos	12.75	496.34	3.14	0.0027	1.40
PAF C-16^S^	up-regulation	C26H54NO7P	pos	14.38	524.37	3.49	0.0214	1.60
LysoPC(18:1/0:0)^p^	up-regulation	C26H52NO7P	neg/pos	13.22	580.3611/522.3582	2.23	0.0049	4.71
LysoPC(18:0/0:0)^p^	up-regulation	C26H54NO7P	neg/pos	14.77	582.4761/524.3715	3.29	0.0051	8.61
LysoPC(16:1(9Z))^p^	up-regulation	C24H48NO7P	pos	11.48	494.32	2.7	0.0086	4.12
LysoPC(18:2/0:0)^p^	up-regulation	C26H50NO7P	pos	11.98	520.34	2.81	0.0158	3.33
LysoPC(18:3/0:0)^p^	up-regulation	C26H48NO7P	pos	12.74	518.32	3.73	0.0046	3.68
LysoPC(14:0/0:0)^p^	up-regulation	C22H46NO7P	pos	10.73	468.31	6.36	0.0081	3.35
LysoPC(20:2/0:0)^p^	up-regulation	C28H54NO7P	pos	13.74	548.37	2.24	0.0124	1.52
LysoPC(20:3/0:0)^p^	up-regulation	C28H52NO7P	pos	13.14	546.35	2.23	0.0146	2.26
LysoPC(20:4/0:0)^p^	up-regulation	C26H52NO7P	pos	11.93	544.34	3.27	0.0073	2.89
LysoPC(22:6/0:0)^p^	up-regulation	C30H50NO7P	pos	11.86	568.34	3.78	0.0079	1.97
LysoPC(15:0)^p^	up-regulation	C23H48NO7P	pos	11.72	482.32	3.68	0.0083	2.88
LysoPC(17:0)^p^	up-regulation	C25H52NO7P	pos	13.36	510.36	3.01	0.0101	5.02
LysoPE(16:0/0:0)^S^	up-regulation	C21H44NO7P	pos	12.65	454.29	2.44	0.0047	2.90
LysoPE(20:4/0:0)^p^	up-regulation	C25H44NO7P	pos	11.87	502.29	2.18	0.0147	3.98
LysoPE(22:6/0:0)^p^	up-regulation	C27H44NO7P	pos	11.80	526.29	2.52	0.0223	2.02
LysoPE(16:1/0:0)^p^	up-regulation	C21H42NO7P	pos	11.20	452.28	2.43	0.0165	1.75
LysoPE(18:1/0:0)^p^	up-regulation	C23H46NO7P	neg	13.07	478.48	2.04	0.0000	2.67
LysoPE(22:1/0:0)^p^	up-regulation	C27H54NO7P	pos	14.11	536.37	2.93	0.0142	2.34
Arachidonic acid^S^	up-regulation	C20H32O2	neg	17.15	303.20	7.16	0.0200	1.05
Linoleic acid^S^	up-regulation	C18H32O2	neg	16.55	279.23	5.03	0.0085	6.57
Palmitic acid^S^	up-regulation	C16H32O2	neg	18.02	281.18	2.61	0.0028	2.20
Oleic acid^S^	up-regulation	C18H34O2	neg	17.72	255.45	4.21	0.0009	3.28

pos and neg represented positive mode and negative mode respectively; s Lipids that were confirmed with authentic standards; p Lipids that putatively annotated and matched the fragmentation pattern with the database.

## Supplementary Materials

The following are available online at http://www.mdpi.com/1999-4915/11/1/73/s1, Table S1: Gradient elution program applied for UPLC-MS analysis, Figure S1: OPLS-DA model validation and permutation test. (a) The cross-validated parameters (R2X = 0.83, R2Y = 0.98, Q2 = 0.97) are satisfactory for the OPLS-DA model; (b) The permutation test (100 times) indicates that the validated model is acceptable, Figure S2: The MS/MS mass spectra and predicted structures with expected fragmentation profiles of the 5 lipids in cell lysate: (A) Linoleic acid; (B) LysoPC(16:0/0:0); (C) LysoPE(16:0/0:0); (D) Arachidonic acid; and (E) PAF C-16.
