# Supplementary material for: Characterization of the Lipidomic Profile of Human Coronavirus-Infected Cells: Implications for Lipid Metabolism Remodeling upon Coronavirus Replication
Source: Viruses. 2019 Jan 16;11(1):73. doi: 10.3390/v11010073 (PMC6357182; doi:10.3390/v11010073)
Supplement: Supplementary file 1 [file viruses-11-00073-s001.pdf]

Table S1. Gradient elution program applied for UPLC-MS analysis.

Time(min)	Flow rate (ml/min-1)	Mobile phase A(%)	Mobile phase B(%)	Curve
Initial	0.4	99.5%	0.5%	6
1	0.4	99.5%	0.5%	6
5	0.4	65%	35%	6
23	0.4	0.5%	99.5%	6
26.5	0.4	0.5%	99.5%	6
27.5	0.4	99.5%	0.5%	6
30	0.4	99.5%	0.5%	6

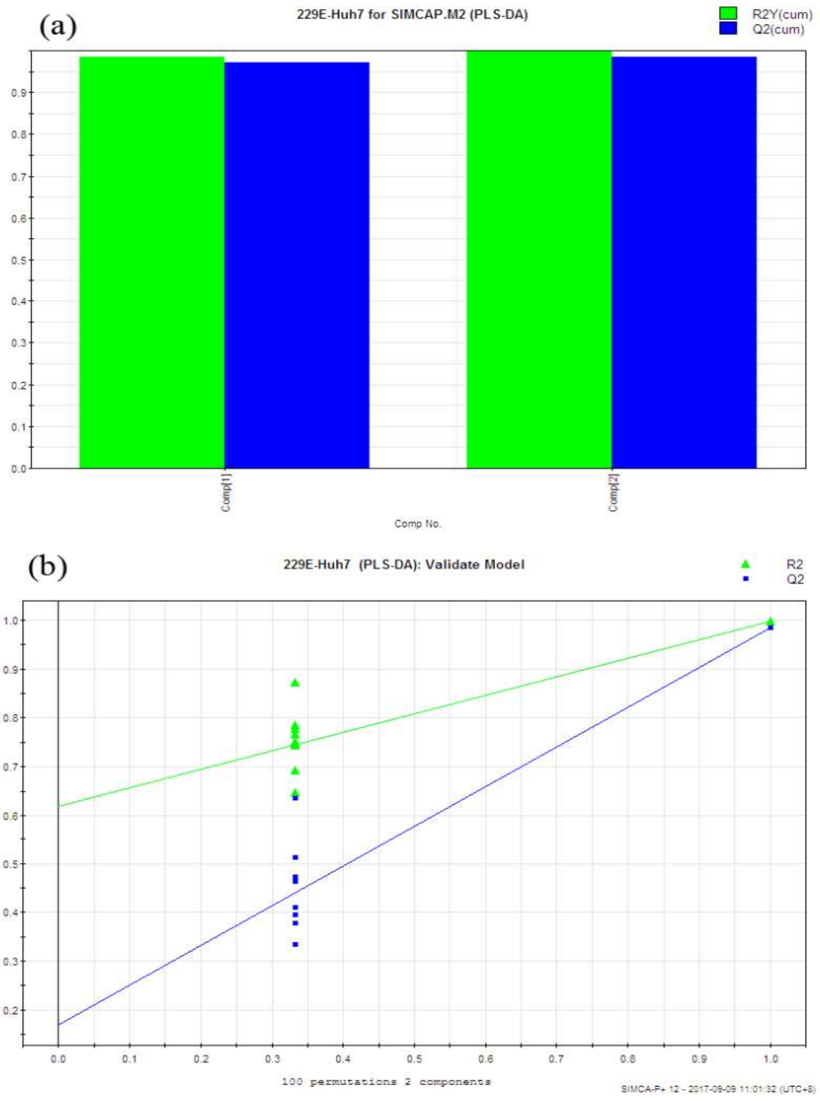

Figure S1. OPLS-DA model validation and permutation test. (a) The cross-validated parameters (R^2X=0.83, R^2Y=0.98, Q^2=0.97) are satisfactory for the OPLS-DA model. (b) The permutation test (100 times) indicates that the validated model is acceptable.

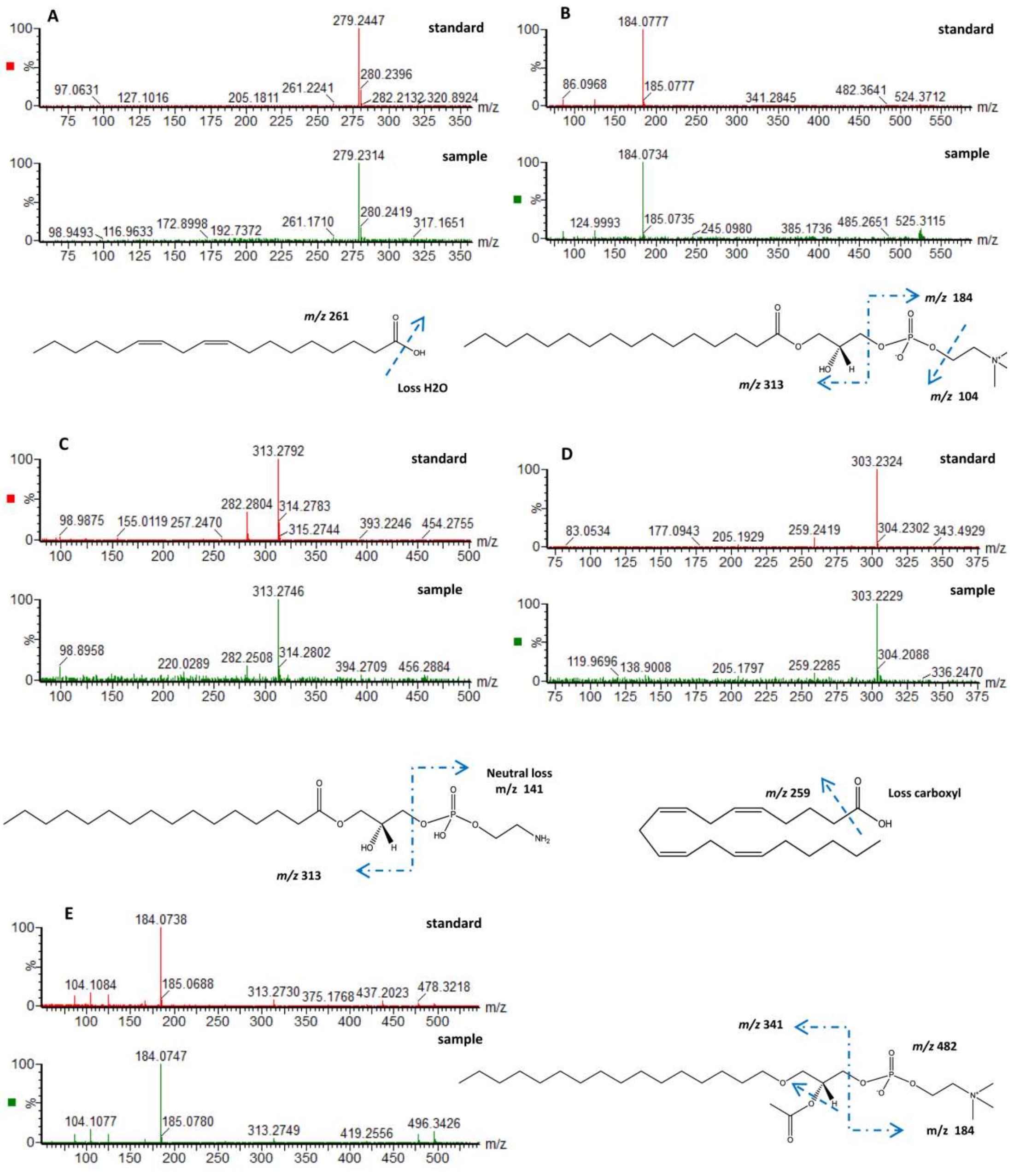

Figure S2. The MS/MS mass spectra and predicted structures with expected fragmentation profiles of the five representative lipids in cell lysate: (A) Linoleic acid; (B) LysoPC(16:0/0:0); (C) LysoPE(16:0/0:0); (D) Arachidonic acid; and (E) PAF C-16.
